# Bidirectional Ventricular Tachycardia in a Postoperative Patient

**DOI:** 10.1016/j.cjco.2024.04.001

**Published:** 2024-04-06

**Authors:** Stephen A. Duffett, Marko Balan, Frédéric L. Paulin, Sean P. Connors

**Affiliations:** a Division of Cardiology, Faculty of Medicine, Memorial University of Newfoundland, St. John’s, Newfoundland, Canada; b Division of Critical Care, Faculty of Medicine, Memorial University of Newfoundland, St. John’s, Newfoundland, Canada

**Bidirectional ventricular tachycardia (VT) classically is associated with digoxin toxicity, catecholaminergic polymorphic ventricular tachycardia** **, and Andersen-Tawil syndrome.** **^1^** **We report a case of bidirectional VT in a postoperative patient with *TMEM43* arrhythmogenic cardiomyopathy** **with a probable diagnosis of stress-induced cardiomyopathy. Despite a documented VT rate that exceeded the detect rate on the patient’s** **implantable cardioverter-defibrillator** **, no therapy was delivered by the device, owing to failure to satisfy consecutive tachycardia intervals, given the varying cycle lengths, due to the alternating QRS morphology characteristic of bidirectional VT.**

A 63-year-old woman with a history of *TMEM43* arrhythmogenic cardiomyopathy (AC) was seen on postoperative day 5 following left thoracotomy and left lower lobectomy for resection of a squamous cell carcinoma. The postoperative course was complicated by hypoxemic respiratory failure requiring high-flow nasal oxygenation to maintain adequate oxygen saturation. On postoperative day 5, she had sudden onset of a wide-complex tachycardia noted on telemetry. A 12-lead electrocardiogram (ECG) of her arrhythmia is shown in Figure 1.

**Figure 1:**
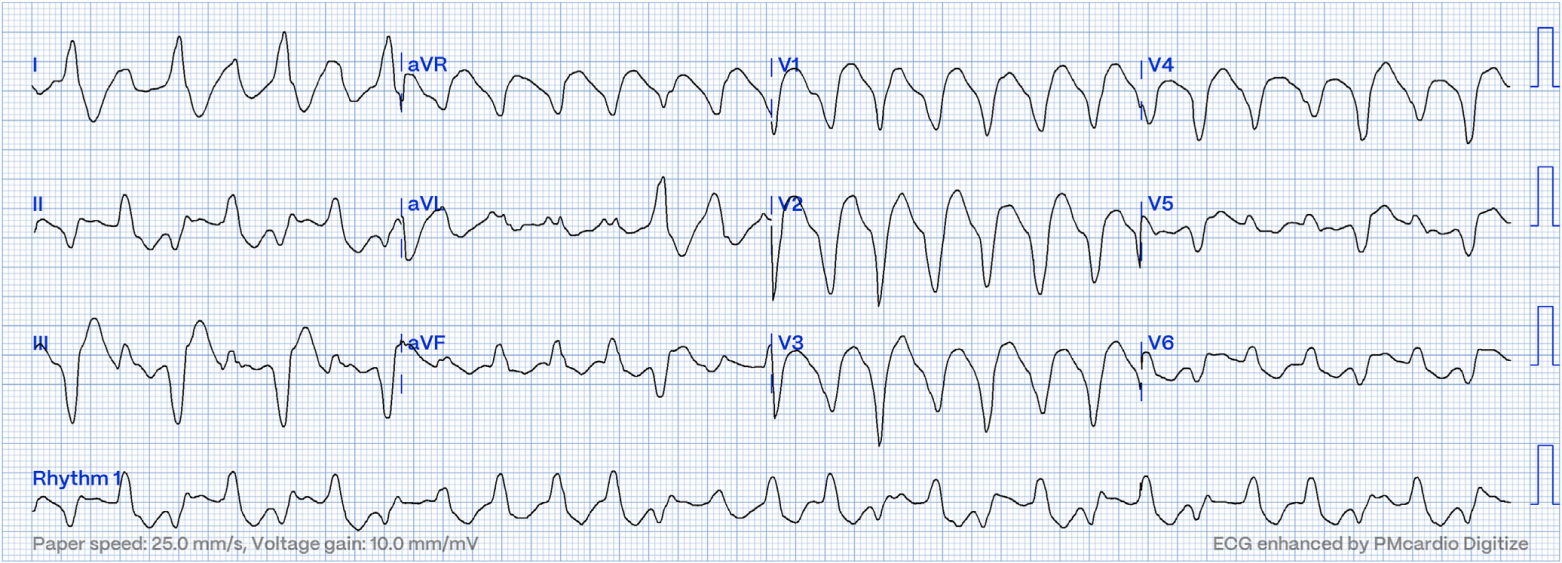
Twelve-lead electrocardiogram (ECG) demonstrating bidirectional ventricular tachycardia (VT). The original ECGs were standardized and digitized using the PMcardio app (Powerful Medical; Bratislava, Slovakia).

The rhythm was determined to be ventricular tachycardia (VT) with frequent periods of alternating frontal plane axis consistent with bidirectional VT. The patient was stable hemodynamically during this episode. She was taking amiodarone 400 mg daily, and sotalol 80 mg twice daily, chronically, for a history of refractory VT requiring device therapy, which was continued in the hospital. Other than these 2 medications, no antiarrhythmic medications had been given. Digoxin had not been used prior to this event. The QTc interval in sinus rhythm on ECG the same day as the VT ECG was 447 ms. A preoperative transthoracic echocardiograph showed mildly decreased left ventricular systolic function, with mid-distal inferolateral and inferoseptal wall hypokinesis, and mild right ventricular dilatation with lower limits of right ventricular systolic function. Right ventricular systolic pressure was estimated as normal, and no significant valvular dysfunction was present. These findings were not significantly different from those of a prior ECG in 2020. An electrolyte panel demonstrated a potassium level of 4.2 mmol/L (normal: 3.5-5.0 mmol/L), a magnesium level of 0.86 mmol/L (normal: 0.66-1.07 mmol/L), and a corrected calcium level of 2.4 mmol/L (normal: 2.15 – 2.55 mmol/L). This episode of tachycardia resolved spontaneously, and she converted back to sinus rhythm with baseline repolarization/T wave abnormalities. No ischemic symptoms or new ECG changes suggested ischemia.

The patient was transferred to the coronary care unit, where she received intravenous amiodarone infusion and treatment for pulmonary edema. On device interrogation, the VT episode was recorded in the monitor zone with a duration of 31 minutes and 50 seconds. No anti-tachycardia pacing (ATP) or defibrillation therapy was delivered by her implantable cardioverter defibrillator (ICD) during this episode. The lower therapy zone of the device was set to 28 intervals at 370 ms (162 beats per minute [bpm]). The upper therapy zones were 320 ms (188 bpm) VT via ventricular fibrillation and 260 ms (231 bpm) for 30/40 intervals. The lower therapy zone was decreased to 410 ms (146 bpm) following this episode. The patient was monitored in the cardiac care unit following this episode, with no recurrence of VT, and was transitioned back to oral amiodarone and sotalol. Follow-up myocardial perfusion imaging in the hospital did not demonstrate any evidence of reversible ischemia. A postoperative echocardiogram demonstrated a new finding of a dyskinetic apex, which resolved on a follow-up study, suggestive of stress-induced (Takotsubo) cardiomyopathy. The patient was discharged in stable condition following the usual postoperative care for thoracic surgery.

## Discussion

Classically, bidirectional VT is associated with digoxin toxicity, CPVT, and Andersen-Tawil syndrome.^1^ The condition has also been described, rarely, in myocarditis, hypokalemic periodic paralysis, and herbal aconitine poisoning.^1^ Other rare reports have been made of bidirectional VT in dilated cardiomyopathy, metastatic cardiac tumours, acute ischemia, and active cardiac sarcoidosis.^1^, ^2^, ^3^, ^4^ Bidirectional VT is not an arrhythmia typically associated with AC. However, a case published in 2015 described a patient with phenotypic features of both AC and CPVT who had bidirectional VT. This patient was genotype-negative, with no pathogenic variant identified on genetic testing for CPVT or AC.^5^ Bidirectional VT also was recently described in a patient with stress-induced (Takotsubo) cardiomyopathy following multiple traumatic injuries and methamphetamine use.^4^ This patient had subsequent recovery of the apical wall-motion abnormality initially noted on echocardiogram.

Our patient had a primary prophylaxis defibrillator implanted in 2002 for a manifest phenotype with the high-risk *p.S358L TMEM43* variant. She had received multiple appropriate shocks for VT and ventricular fibrillation over long-term follow-up. She was started on sotalol in 2004, and was later transitioned to amiodarone in 2016 for recurrent episodes of ventricular arrhythmia. At the most-recent follow-up, in November of 2023, she was taking both amiodarone and sotalol for recurrent episodes of refractory ventricular arrhythmia.

In this case, the cause of bidirectional VT was felt to be most likely secondary to stress-induced cardiomyopathy, although whether the underlying AC contributed to its development is unclear. In addition, the use of amiodarone and sotalol in combination possibly could have contributed to development of this arrhythmia. However, these medications have not been attributed previously to this type of VT, and the patient had been on stable chronic therapy prior to this event. Only single reports of bidirectional VT in AC and stress cardiomyopathy have been made; both were present in this patient.

**Figure 2:**
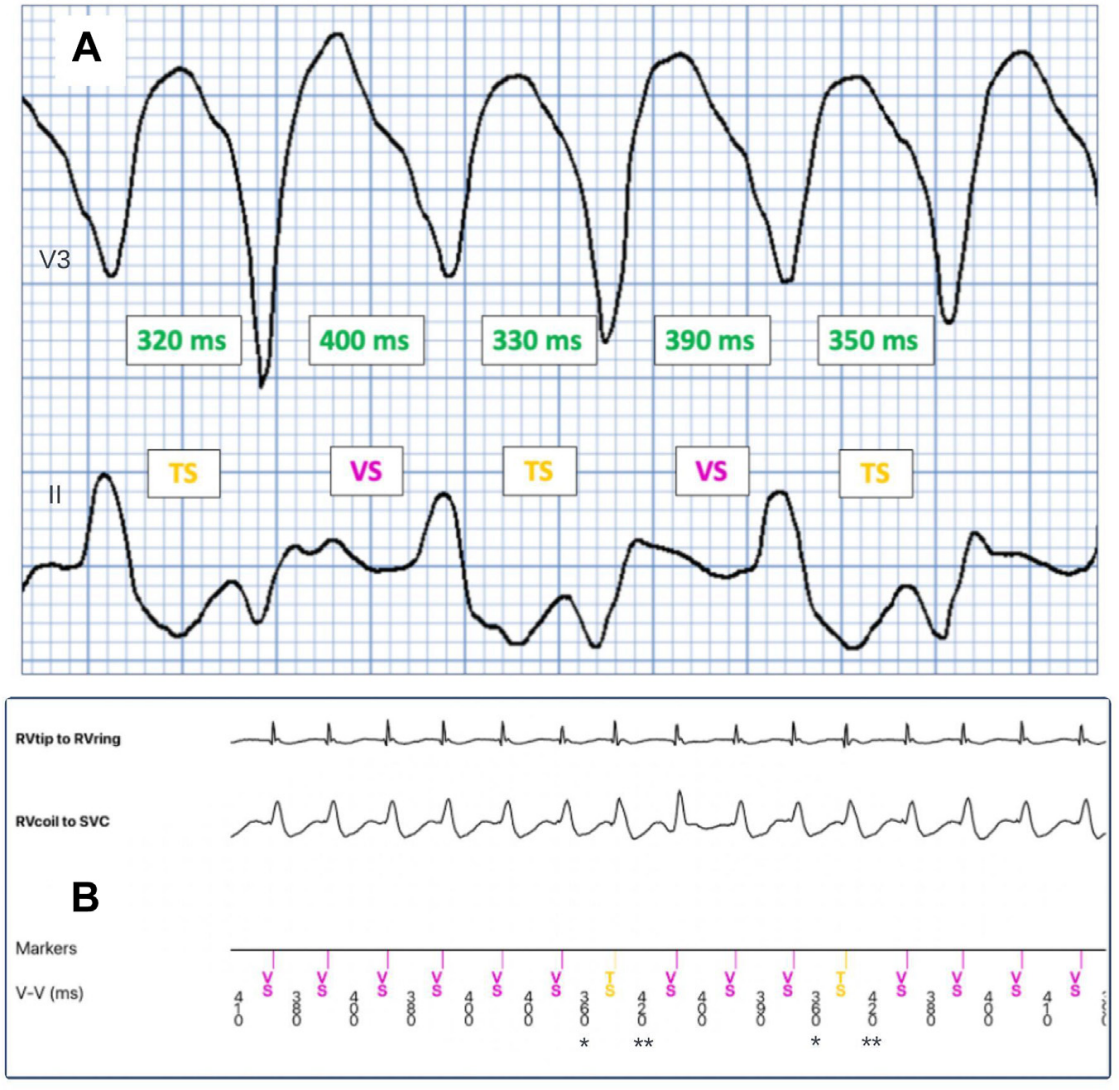
(**A**) Varying beat-to-beat cycle length during bidirectional ventricular tachycardia (VT). (**B**) Short (**asterisk**) and long (**double asterisks**) interval pairs noted during VT. TS, indicates a tachycardia-zone interval (tachycardia-sensed); VS, indicates an interval outside the tachycardia zone (ventricular-sensed). RV, right ventricular; SVC, superior vena cava.

An additional interesting aspect of this case is its implications for programming of the ICD for tachycardia therapy. The rate of the VT on the ECG shown in Figure 1 is an average of 162 bpm. This rate happened to be the exact rate of the lower therapy zone of the ICD, which resulted in some intervals falling below the detect rate. VT at or below the detect rate of a device is a common issue. However, in this case, the presence of alternating QRS morphologies added an additional challenge for tachycardia detection that is interesting to consider. Some tachycardia therapy zones are reliant on consecutive counts of tachycardia intervals, whereas others are probabilistic. The lower zone for this device was set to 28 consecutive intervals, which means that 28 consecutive intervals greater than 162 bpm need to be seen to trigger therapy. A single interval outside of the tachycardia therapy zone will reset the counter. Although the tachycardia had an average rate close to that of the lower therapy zone, beat-to-beat variations in the morphology produced uneven cycle-length intervals, with some intervals satisfying the detection requirement, and others falling out of the detection range, as illustrated in Figure 2A.

As demonstrated in Figure 2A, the QRS complexes demonstrate short intervals followed by long intervals. The average of these intervals is 358 ms (168 bpm). If this pattern were a regular monomorphic tachycardia at 168 bpm, all intervals would have satisfied conditions for treatment, with the patient’s current programming (lower zone of 370 ms/162 bpm). However, only intervals labelled tachycardia-sensed) fall into tachycardia therapy zones, whereas ventricular-sensed intervals fall outside these zones. This cycle-length variation is due to the alternating QRS morphology. Given that the episode lasted for 32 minutes, and the device suspends recording for part of this record, the exact electrogram annotation on the device corresponding to our 10-second ECG was not available. However, part of the episode was recorded on the device and confirms intermittent short and long interval pairs corresponding to bidirectional beats, as shown in Figure 2B. Typically, bidirectional VT shows frontal axis alterations occurring beat-to-beat. In this case, the rhythm appears to be intermittently monomorphic, with periods of sustained bidirectionality (Fig. 1). The reason for this pattern is unclear, but it may be due to simultaneous and competing bidirectional VT from triggered activity, and re-entrant VT due to AC. In the part of the episode shown in Figure 2B, the cycle length did slow considerably, becoming lower than the lower therapy zone throughout the 32 minutes. However, the episode may have been treated by the device earlier if the alternating QRS did not result in cycle-length variations. The average rate of the tachycardia recorded by the ICD throughout its duration was 164 bpm. The lower therapy zone of the device was reduced from 162 bpm to 146 bpm following this event.

In summary, this case describes the rare finding of bidirectional VT in a patient with AC seen postoperatively who likely also had a diagnosis of stress-induced cardiomyopathy. Despite the average rate of the tachycardia exceeding the lower therapy zone of the defibrillator, no therapy was delivered by the device. Beat-to-beat alteration in the QRS morphology produced a varying cycle length that prevented appropriate recognition and treatment of VT by the ICD. This case demonstrates how bidirectional VT may not trigger device therapy in an ICD, despite meeting the rate criteria for a tachycardia zone. Therapy zones that require consecutive intervals to satisfy detection requirements may miss detection of this rare arrhythmia.

**Novel Teaching Points**

• Bidirectional VT is a rare arrhythmia with a relatively short list of possible causes.
• Bidirectional VT in this case may be secondary to AC or stress-induced cardiomyopathy, both of which have been reported rarely.
• ICD therapy may be withheld inappropriately due to failure to meet consecutive interval requirements when the QRS morphology is alternating beat-to-beat in bidirectional VT.

## References

[1] Sonmez O., Gul E.E., Duman Ç. (2009). Type II bidirectional ventricular tachycardia in a patient with myocardial infarction. J Electrocardiol.

[2] Benjamin M.M., Hayes K., Field M.E., Scheinman M.M., Hoffmayer K.S. (2017). Bidirectional ventricular tachycardia in cardiac sarcoidosis. J Arrhythmia.

[3] Norwood D.A., Dominguez L.B., Dominguez R.L., Winders W.T. (2019). Bidirectional ventricular tachycardia in a women with dilated cardiomyopathy: a case report. Adv J Emerg Med.

[4] Schreiber A., Gardner M., Sossou C., Greene N., Ahsan C. (2022). Bidirectional ventricular tachycardia with stress-induced cardiomyopathy. Case Rep Cardiol.

[5] Patel H., Shah P., Rampal U., Shamoon F., Tiyyagura S. (2015). Arrythmogenic right ventricular dysplasia/cardiomyopathy (ARVD/C) and cathecholaminergic polymorphic ventricular tachycardia (CPVT): a phenotypic spectrum seen in same patient. J Electrocardiol.

